# Supplementary material for: Correlator convolutional neural networks as an interpretable architecture for image-like quantum matter data
Source: Nat Commun. 2021 Jun 23;12:3905. doi: 10.1038/s41467-021-23952-w (PMC8222395; doi:10.1038/s41467-021-23952-w)
Supplement: Supplementary file 1 — Supplementary Information [file 41467_2021_23952_MOESM1_ESM.pdf]

Supplementary Information

Cole Miles,[1] Annabelle Bohrdt,[2,3,4] Ruihan Wu,[5] Christie Chiu,[2,6,7] Muqing Xu,[2] Geoffrey Ji,[2] Markus Greiner,[2] Kilian Q. Weinberger,[5] Eugene Demler,[2] and Eun-Ah Kim[1,*]

[1] *Department of Physics, Cornell University, Ithaca, NY 14853, USA*
[2] *Department of Physics, Harvard University, Cambridge, MA 02138, USA*
[3] *Department of Physics and Institute for Advanced Study, Technical University of Munich, 85748 Garching, Germany*
[4] *Munich Center for Quantum Science and Technology (MCQST), 80799 München, Germany*
[5] *Department of Computer Science, Cornell University, Ithaca, NY 14853, USA*
[6] *Department of Electrical Engineering, Princeton University, Princeton, NJ 08540, USA*
[7] *Princeton Center for Complex Materials, Princeton University, Princeton, NJ 08540, USA*

I. SUPPLEMENTARY NOTE 1: TRAINING DETAILS

a. Training data generation Equilibrium snapshots of both models – π-flux theory and geometric string theory – can be sampled straightforwardly, as discussed in [1] and [2]. We here briefly summarize the corresponding sampling techniques.

Geometric string theory only makes a statement about how the doped model deviates from half-filling. The spin-background at half-filling here is given by sampling snapshots of the Heisenberg model at finite temperature using quantum Monte Carlo techniques. We sample snapshots for a 40×40 system with periodic boundary conditions and then cut out a 16×16 observation region from each snapshot. For a given doping level, we then insert the corresponding number of holes into each snapshot at random positions, where holes cannot sit on the same site. The geometric string theory provides a distribution of string lengths for a given temperature [2, 3]. For each hole, we thus sample a string length from the string length distribution and move the hole by hand for a corresponding number of sites through the spin background in random directions while displacing the spins along its path.

Snapshots from π-flux theory are generated using standard Metropolis Monte Carlo sampling of the Gutzwiller projected thermal density matrix of the mean-field Hamiltonian [4]

$$\begin{aligned}\hat{\mathcal{H}}_{\mathrm{MF}} = &-\frac{1}{2}J^* \sum_{\mathbf{i}\in A}\sum_{\sigma}\left(e^{i\theta_0}\hat{c}^\dagger_{\mathbf{i},\sigma}\hat{c}_{\mathbf{i}+\mathbf{x},\sigma} + e^{-i\theta_0}\hat{c}^\dagger_{\mathbf{i},\sigma}\hat{c}_{\mathbf{i}+\mathbf{y},\sigma} + h.c.\right)\\ &-\frac{1}{2}J^* \sum_{\mathbf{i}\in B}\sum_{\sigma}\left(e^{-i\theta_0}\hat{c}^\dagger_{\mathbf{i},\sigma}\hat{c}_{\mathbf{i}+\mathbf{x},\sigma} + e^{i\theta_0}\hat{c}^\dagger_{\mathbf{i},\sigma}\hat{c}_{\mathbf{i}+\mathbf{y},\sigma} + h.c.\right).\end{aligned} \tag{1}$$

Here, $\mathbf{i} \in A(B)$ denotes lattice sites $\mathbf{i}$ which are part of the A(B) sublattice and $\hat{c}^{(\dagger)}_{\mathbf{i},\sigma}$ is the annihilation (creation) operator of a fermion with spin σ. The mean-field Hamiltonian describes a system with staggered flux $\pm\Phi = \pm 4\theta_0$ and effective hopping amplitude J^*. In particular, we consider π-flux states with $\theta_0 = \pi/4$. We simultaneously sample the occupation in momentum and real space. The real and momentum space configurations are denoted as $|\tilde{\alpha}_{\mathbf{r}}\rangle$ and $|\alpha_{\mathbf{k}}\rangle$, respectively. In momentum space, the two spin species are treated separately, such that two fermions of opposite spin can occupy the same momentum state. In real space, two fermions with opposite spin cannot occupy the same site, thus directly implementing the Gutzwiller projection. In any given real space configuration $|\tilde{\alpha}_{\mathbf{r}}\rangle$, each site is therefore either empty or occupied with a spin up or a spin down fermion. The mean field Hamiltonian (1) can be readily diagonalized in momentum space. For each momentum space configuration $|\alpha_{\mathbf{k}}\rangle$, we thus directly obtain an energy $E(\alpha_{\mathbf{k}})$ and from that the corresponding thermal weight. We use the Metropolis Monte Carlo algorithm [5] to sample Gutzwiller projected real space snapshots $|\tilde{\alpha}_{\mathbf{r}}\rangle$ according to the probability distribution

$$p_\beta(\tilde{\alpha}_{\mathbf{r}}, \alpha_{\mathbf{k}}) = Z^{-1} e^{-\beta E(\alpha_{\mathbf{k}})} |\langle \tilde{\alpha}_{\mathbf{r}} | \alpha_{\mathbf{k}} \rangle|^2. \tag{2}$$

The overall energy scale J^* of this model is treated as a free parameter which is fit so that the nearest-neighbor spin correlators match with those of geometric string theory at half-filling. We sample snapshots of size 16×16 with periodic boundary conditions.

---

* eun-ah.kim@cornell.edu

Our dataset consists of 24075 total sampled snapshots, with 18067 of them used as the training set, 2008 as the validation set, and 4000 as the test set. The validation set is not seen by any of the networks during training, but is used for us to gauge generalization of the models and to set hyperparameters. The test set is neither seen by us or the model until after the final model is chosen.

b. Training Procedure As described in the main text, training is done in two phases. The first is done in PyTorch [6], in which the full model, including both the convolutional filters and the logistic classifier, is trained using the `ADAM` optimization algorithm. The resulting model from this process can be used as-is for classification. However, for an interrogation of which features are most important, regularization paths are produced in a second phase. Here, the convolutional filters are held fixed and only the back logistic classifier is trained multiple times over a wide range of L1 λ coefficients. This phase is done in Scikit-Learn [7] due to its extremely efficient logistic regression routines.

Optimizer	`Adam`
Adam β_1	0.9
Adam β_2	0.999
Adam ϵ	1×10^{-8}
Learning Rate	0.005
LR Schedule	`CosineAnnealingLR`
Batch Size	1024
L1 Coefficient, γ	0.005
Epochs	1000
Number of Filters	2
Seed	4444

Supplementary Table 1: **Hyperparameters used to train the CCNN model presented in the main text.** The random seed used is included to allow full reproducibility using the public code.

During the first phase, the optimization process attempts to minimize the loss which we write in the general multi-class form:

$$L_{\text{train}}(y, \hat{y}) \equiv -\sum_i y_i \log \hat{y}_i + \gamma \sum_\alpha ||f_\alpha||_1 \tag{3}$$

where the first term is the standard cross-entropy loss used for classification tasks, and the second term is an L1 (or LASSO) regularization which has the effect of driving the unimportant components of the convolutional weights f_α to zero.

To allow for simple interpretation of the resulting filters, we strictly limit the f_α to take on only positive values. This can either be done by replacing the f_α in place with their absolute value after each gradient update, or by simply taking the absolute value every time a forward pass is done. This was found to incur a $\sim 2\%$ accuracy loss, which we find acceptable in order for easier interpretation. In contrast, forcing the filter weights to be positive seems to entirely halt the learning process for traditional CNN architectures.

The loss during the second phase is similar:

$$L_{\text{path}}(y, \hat{y}) \equiv -\sum_i y_i \log \hat{y}_i + \lambda ||\boldsymbol{\beta}||_1 \tag{4}$$

with the notable difference that the LASSO regularization is now being applied to the logistic weights rather than the convolutional filters. A LASSO regularization path is formed by minimizing this loss repeatedly while varying the value of λ, and plotting the learned model coefficients $\beta_\alpha^{(n)}$ as a function of λ. The L1 loss plays a special role in forming this regularization path, as its geometric properties tend to drive coefficients completely to zero rather than just a small value [8]. However, there do exist alternate algorithms which also could be used, which are either more efficient [9] or use alternate loss functions with different properties [10].

We utilize a BatchNorm [11] layer intermediate between the nonlinear convolutions and the logistic classifier, without the additional learnable affine transformation typically used as we find these introduce additional complexity without much benefit for our problem. During training, this layer simply normalizes the features produced from each minibatch to be zero mean and unit variance to allow for easier classification. During validation, the layer uses exponential running estimates of the mean and variance for normalization rather than the minibatch statistics. We empirically found this layer to be essential to creating a well-performing architecture, with the hypothesis that this is related to the different scales that each order of nonlinear feature tends to exist at. Normalization brings all of the features to the same relative scale, allowing the classifier to have an easier time detecting distributional differences.

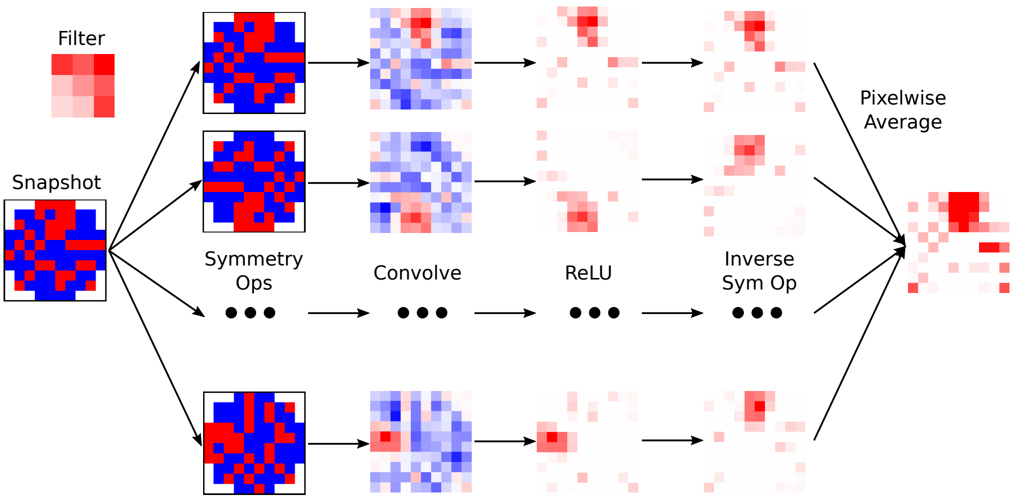

Supplementary Figure 1: **A diagram demonstrating D_4 symmetric convolutions.** This diagram shows how to construct D_4 symmetric convolutions in a traditional CNN architecture. In our CCNN, the interior operations in this process are replaced with our nonlinear convolution operations, but the initial symmetry-slicing and final symmetry-pooling are unchanged.

However, there does exist an unintended interaction between L1 regularization and BatchNorm. Due to the normalization process, the architecture is invariant to an overall scaling of the filter weights. Meanwhile, our intended goal of using L1 was to drive “unimportant” pixels to zero. In a sense, the network can do this for “free” since it can scale the filter weights without any loss in performance. In practice however, we find that the L1 loss still does bias the network towards having lower complexity filters while keeping the overall scale reasonable. However the relationship between the λ parameter and the number of pixels activated is not always simple - sometimes increasing λ will result in more pixels activated. While the interaction between L2 regularization and BatchNorm is well understood [12], the authors of this work are unaware of any similar understanding with L1 regularization. A solution to this issue is still a desired feature.

c. Symmetrization One factor leading to the overparameterization of standard CNNs is that to reach peak accuracy, they need to explicitly learn multiple symmetry-equivalent versions of spin patterns. To achieve the same effect without requiring the duplication of filters, we use a D_4 symmetry-equivariant form of the convolutional operation as introduced in [13]. A visual explanation of the operation as performed in a standard CNN pipeline can be seen in Supplementary Fig. 1.

Modification to suit our architecture is simple, following the steps described in [13] to extend this idea to arbitrary models. Before any operation is applied, a “symmetric slicing” operation is done which stacks extra rotated/flipped copies of the input into the batch dimension. The rest of the operations in the architecture are applied as usual to the entire batch. Then, before feeding the final features into the logistic classifier, a “symmetric pooling” operation applies the correct inverse symmetry operations to each copy of the input, then averages across them. This entire block of operations then forms features which are equivariant to the desired symmetries of the input. If these features are then spatially averaged, they instead form invariants (in which case the aforementioned inverse symmetry operations are not needed). For fair comparison, every model examined in this work had this symmetrization applied.

d. Performance Measurements In Supplementary Fig. 2a, we show the performance of our architecture at various orders to which the model is constructed, compared against a traditional CNN architecture using ReLU as the nonlinearity. (For details, see the “reduced architecture” described in Sec S.V). We also have compared to the much larger architecture of [1], adapted to accept three-channel snapshots as input, though it is difficult to control the overfitting even with strong regularization. Meanwhile, our architecture does not show signs of significant overfitting even in absence of regularization due to its small parameterization. Out of all of our trials, for a fixed number of filters no tested CNN has outperformed our CCNN models on the validation dataset by more than $\approx 1\%$, which disappears if we restrict the CNN filters to be positive as done in the CCNN.

Each curve shown is labeled with the number of filters that model contains; we increase the number of filters as the order of the model decreases to keep the total number of features relatively constant, for a fair comparison. The solid lines show the running-max (over all previous epochs) of the median validation accuracy achieved between five independent training runs on the same train-val split of the data, but with different parameter initializations and batching order, while the shaded regions shown the min-max spread across these models. Note that, to avoid unfairly

biasing the higher-order networks, the models shown here are not trained with the L1 regularization on the filter weights. See, for example, Supplementary Fig. 11(c) for training curve comparisons.

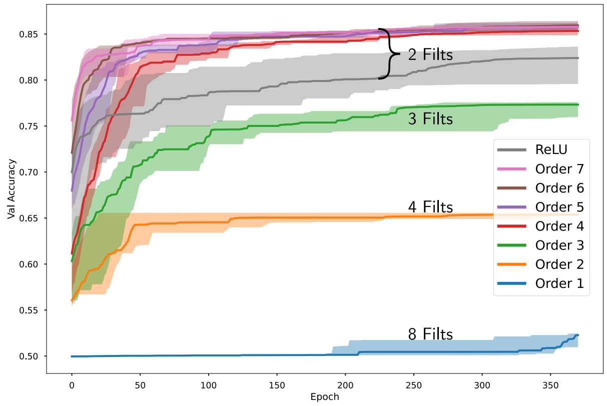

(a) Running-max performance of our architecture compared at different orders we construct the model. We compare against a traditional CNN architecture using ReLU as the nonlinear function. The solid lines indicate the median of five independently-trained models, while the shaded regions show the min-max spread across these models.

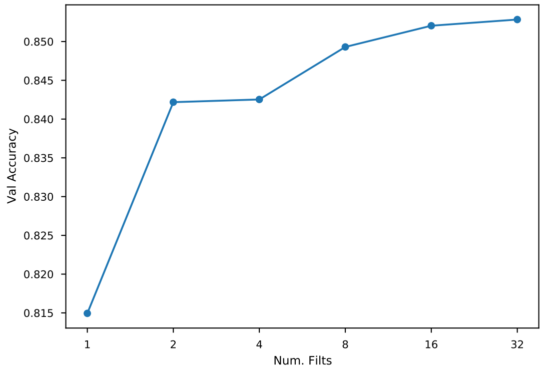

(b) Performance of a fourth-order architecture containing different numbers of convolutional filters.

Supplementary Figure 2: **CCNN performance results**.

We see that the performance of the architecture rapidly increases as a function of the order to which the model is constructed, plateauing past fourth order. At this order, we consistently match performance with traditional CNN architectures, even with only two convolutional filters and dramatically fewer learnable parameters. We find performance plateauing past fourth order to be a general behavior, independent of the number of filters, regularization strength, etc. This indicates that fifth-order and higher correlations provide no new statistically significant information between the two datasets, at least at the snapshot sizes we use.

In Supplementary Fig. 2b, we show the final trained performance of our architecture as a function of the number of filters used. For these measurements, L1 regularization is turned off, as it may prevent additional filters from being used at all. Interestingly, we find that we can get close to optimal performance with just a single convolutional filter, with performance quickly plateauing past this.

We find that the parallel spin and L-shaped patterns shown in the main text are generally robust features learned by the architecture. The "interlocking-L" pattern is extremely robust, with some variant occurring in nearly every trained model. While the parallel spin pattern does not appear in every trained model, it does seem to be the second most common pattern. In Supplementary Fig. 3, we show examples of models trained on the same data with different random seeds controlling the initialization. We note that while the exact filter pattern varies between training runs, the dominant subpatterns tend to match between all of runs, with some alternate local minima.

e. Duplicate Snapshots In principle, finite-size snapshots do not have a "true" assignment to either of the models, as there is a finite probability to sample the snapshot from either distribution. While we do not find any duplicate snapshots between our two model datasets, it is interesting to consider the implications of models whose supports significantly overlap. Consider a collection of models which define probability distributions over the snapshots $\{p_i(\mathbf{x}) | i = 1, \ldots, n\}$. If the dataset consists of an equal number of samples from each class, one can show that the model which minimizes the expected cross-entropy loss in Supplementary Eq. 3 (neglecting the regularization term) then predicts the relative probability to sample $\mathbf{x}$ from each p_i:

$$\hat{y}_i(\mathbf{x}) = \frac{p_i(\mathbf{x})}{\sum_j p_j(\mathbf{x})}. \tag{5}$$

For each sample, predicting the class to be $\arg\max_i \hat{y}_i$ then makes this model into the *Bayes optimal classifier*, which achieves the maximum possible classification accuracy over all possible classifiers [14]. Accuracy here is defined to be the probability of making the correct decision. It is clear that this maximum accuracy is less than 100%, so we can never hope for a perfect classifier, and in practice we can only hope that our training procedure gets us close to

this optimal model. However, in this scenario the probabilities output by the classifier are still meaningful, and we can still uncover information about the relative strength of correlations between all of the distributions.

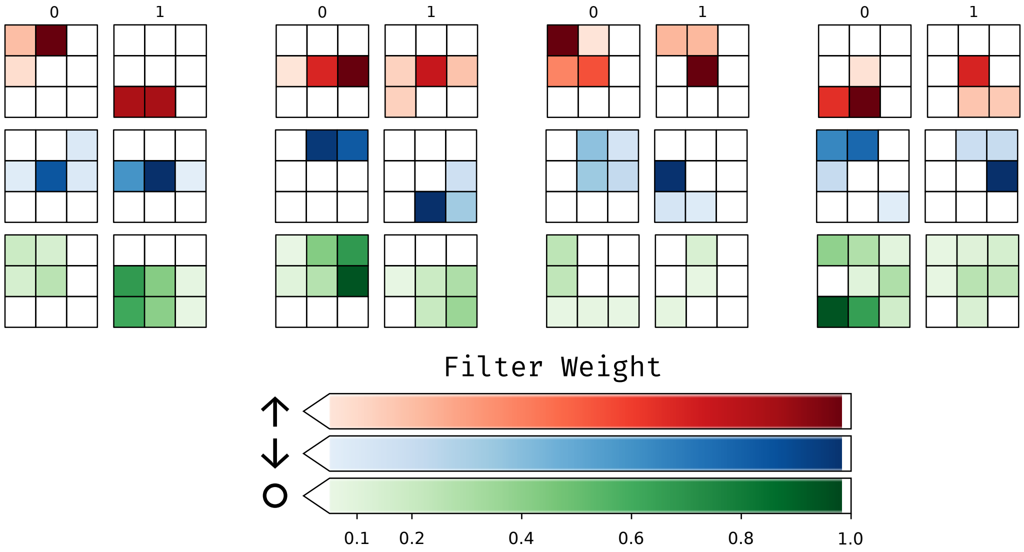

(a) Filters learned from a variety of two-filter models.

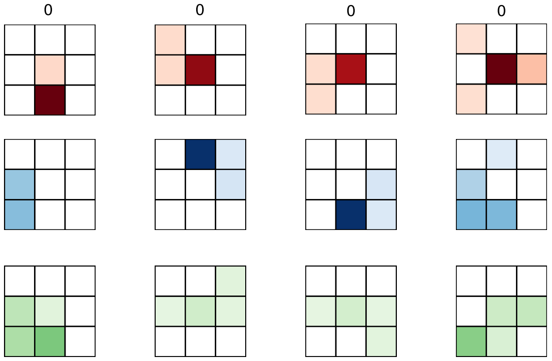

(b) Filters learned from a variety of single-filter models

Supplementary Figure 3: **Examples of filters learned from independently trained models.**

II. SUPPLEMENTARY NOTE 2: OTHER EXAMPLES, ORDER REGULARIZATION

In this section, we provide a few pedagogical examples of applying the CCNN to simpler state discrimination problems, as well as introduce a simple modification to the regularization to simplify regularization paths.

The first example we consider is using CCNNs to identify the presence of antiferromagnetic order in the parent Heisenberg model. To do this, we set up a problem of training a CCNN to discriminate between snapshots sampled from this half-filled, finite-temperature Heisenberg model, and completely random snapshots. The AFM Heisenberg snapshots are generated using the same Quantum Monte Carlo simulations as the doped snapshots investigated in the main text. The random snapshots are sampled such that each site has equal probability of being either spin up or spin down, with the constraint that the total number of spin up sites in each snapshot equals the total number of spin down sites. As a second example, we introduce a mock dataset imitating a stripe-ordered phase. Snapshots are produced by beginning with a perfectly ordered stripe snapshot, then randomly flipping each spin with some probability p. Examples of snapshots sampled from these test distributions are shown in Supplementary Fig. 4.

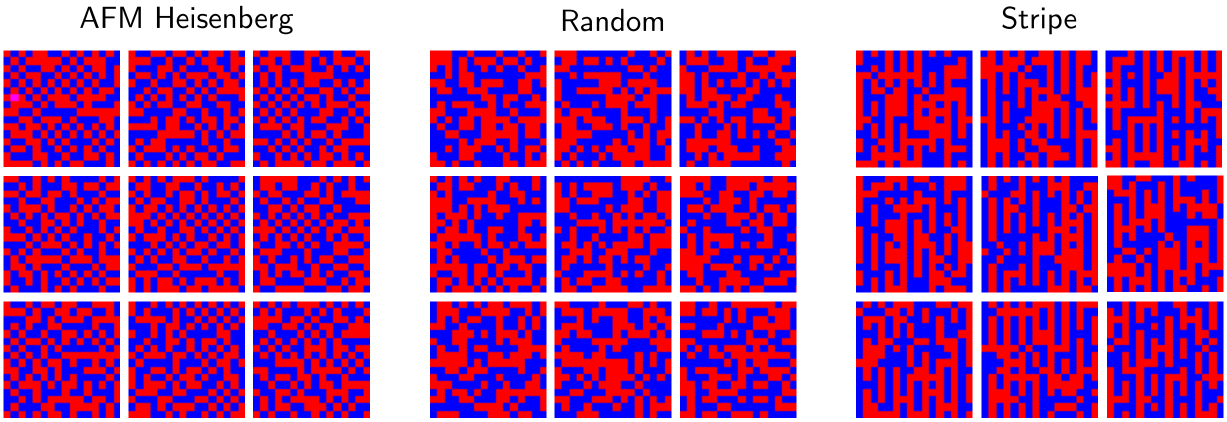

Supplementary Figure 4: **Examples of snapshots from three test distributions.** On left, snapshots sampled from the half-filled antiferromagnetic Heisenberg model. In middle, entirely random spin snapshots constrained to zero total magnetization. On right, snapshots from a mock "stripe-ordered" distribution with $p = 0.3$.

We first train a fourth-order CCNN model to distinguish between the AFM Heisenberg and random snapshots for 100 epochs, and find that a single-filter network easily achieves $> 99\%$ accuracy on both the training and validation datasets. To achieve as simple of a model as possible, we train multiple models of this type, increasing the L1 coefficient on the filter weights, γ (Supplementary Eq. 3), to as large as possible without seeing a drop in performance. This was found to be $\gamma \approx 10$. The final learned filter, and accompanying regularization path can be seen in Supplementary Fig. 5.

We can clearly see from the learned filter that the CCNN is indeed picking up the AFM order present in the Heisenberg snapshots. The regularization path shows this pattern correctly attributed to the Heisenberg model, and furthermore shows that although we trained a fourth order model, two-point correlations of AFM type are sufficient to classify between these distributions with close to 100% accuracy.

Next, we train a CCNN to classify between these stripe ordered snapshots and the random snapshot dataset. Since our mock stripe phase breaks rotational symmetry, we remove rotations from the list of symmetries used in the symmetrization process outlined in the previous section. We again use $\gamma = 10$ and train for 100 epochs. The network rapidly achieves $> 99\%$ accuracy, and results in the learned filter and regularization path shown in Supplementary Fig. 6. Here, it is clear that the network is learning to pick up all two-point spin correlations that are compatible with the stripe pattern.

While this did not occur in the previous examples, it is sometimes the case that regularization paths of CCNNs will show the model using higher-order correlations than one might initially think are relevant. This is because the expectation values of the bare correlators that the CCNN measures are linked to the expectation values of lower-order correlators. For example, in a non-interacting system higher-order correlators can be explicitly decomposed in terms of two-point correlators using Wick contractions. Hence, in some instances these higher-order correlations carry the "same information" as the lower-order correlations, and which one the network chooses to use is somewhat arbitrary. (Though, usually the estimates of these higher correlators are worse and so are unused.)

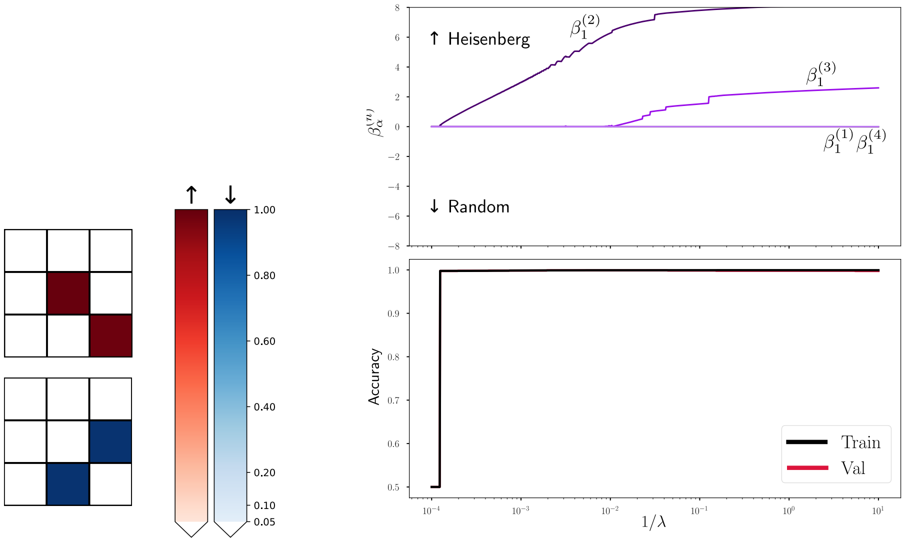

(a) Learned filter. (b) Associated regularization path.

Supplementary Figure 5: **Trained model for the AFM versus random test case.** The filter and regularization paths for a model trained to discriminate between AFM Heisenberg and random snapshots, with filter regularization strength $\lambda = 10.0$.

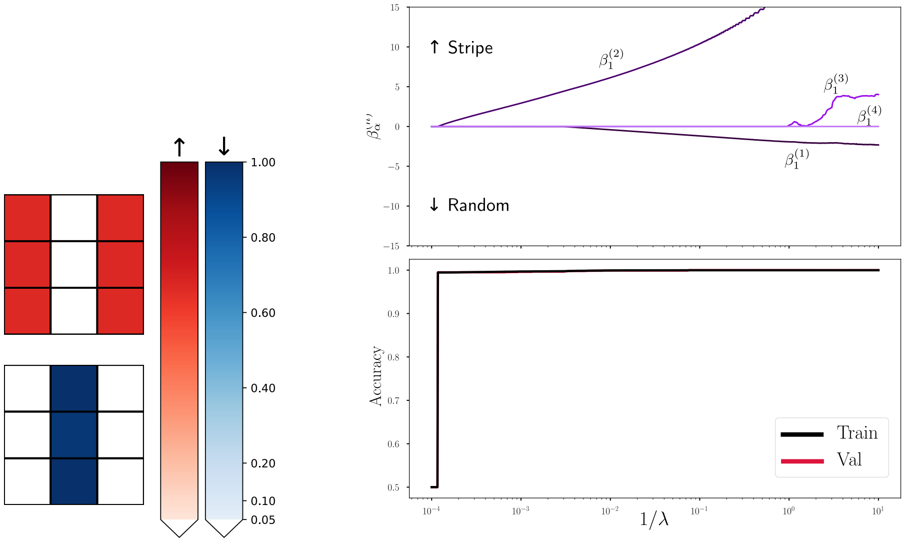

(a) Learned filter. (b) Associated regularization path.

Supplementary Figure 6: **Trained model for the stripe versus random test case.** The filter and regularization paths for a model trained to discriminate between stripe and random snapshots, with filter regularization strength $\lambda = 10.0$.

To break this redundancy, one can modify the loss in Supplementary Eq. 4 to slightly penalize the use of higher-order correlators by making the regularization strength depend on order, $\lambda \to \lambda^{(n)}$:

$$\tilde{L}_{\text{path}}(y, \hat{y}) \equiv -\sum_i y_i \log \hat{y}_i + \sum_{\alpha,n} \lambda^{(n)} |\beta_\alpha^{(n)}| \tag{6}$$

In practice, to break redundancies we only need to make $\lambda^{(n)}$ slightly vary with n. For example, we have found the choice $[\lambda^{(1)}, \lambda^{(2)}, \lambda^{(3)}, \lambda^{(4)}] = [\lambda, 1.1\lambda, 1.2\lambda, 1.3\lambda]$ for each fixed λ to be sufficient for all problems tested. We note that

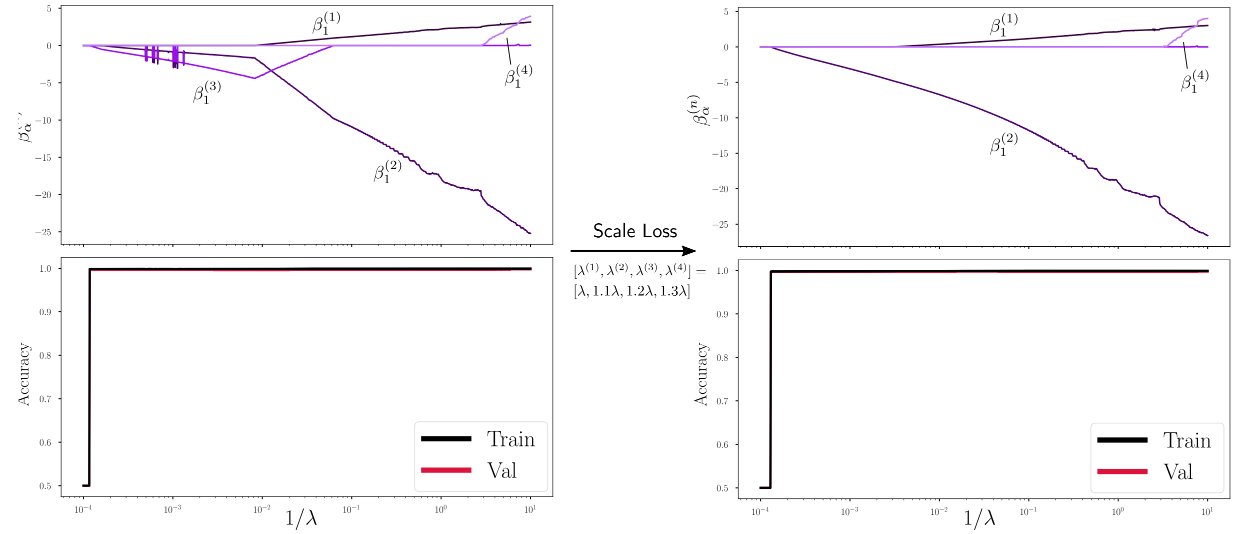

Supplementary Figure 7: **Demonstration of order regularization to resolve redundancies.** On the left, an example of a regularization path (unrelated to the filter shown in Supplementary Fig. 6) struggling to pick between two orders which contain the same information, evidenced by the small gap between their activation and noisy trajectories resulting from convergence issues. On the right, we show how gently scaling the regularization loss coefficient can break this redundancy while maintaining the same classification performance.

including this sort of loss on the models shown in the main text was not found to produce any meaningful qualitative change in the regularization paths – this indicates that there, the higher-order correlators are indeed developing a nonzero connected component which is critical in the decision-making. However, we show in Supplementary Fig. 7 that in other problems, such as these examples, occasionally a regularization path will struggle to pick between different orders when using the original loss function. With the change outlined above, we see that we break this redundancy, resulting in the model choosing the lowest-order distinguishing correlator.

This redundancy-breaking is also used in Supplementary Note 6 where we examine applying the CCNN architecture to spin-unresolved experimental data. As a final note, in these pedagogical examples, for interpretability we would generally would like to obtain filters which measure correlators indicative of the target theory (rather than a relative indication of random noise). While this was not done in the above examples, this can be easily enforced by forcing the $\beta_\alpha^{(n)}$ coefficients to be positive during training.

III. SUPPLEMENTARY NOTE 3: PROOF OF EQ. 3

For simplicity, in this section we will use subscripts to represent spatial indexing, and forgo a channel index. I.e. S_i represents the value of the input at site i. Since information from different filters is never mixed, it is sufficient to consider this operation with a single filter f_a where a indexes the sites within the convolutional window. Furthermore, define $x_a \equiv f_a S_{i+a}$. Our goal is then to show, with the definition

$$C^{(n)} \equiv \sum_{a_1 \neq \ldots \neq a_n} \prod_{j=1}^{n} x_{a_j}, \tag{7}$$

that the following recursive formula holds:

$$C^{(n)} = \frac{1}{n} \sum_{l=1}^{n} (-1)^{l-1} \left(\sum_a x_a^l \right) C^{(n-l)}, \tag{8}$$

with the definition that $C^{(0)} = 1$.

This can be confirmed by direct substitution and unwrapping the sum term-by-term. The $l = 1$ term of the sum reads

$$(l=1) \qquad \left(\sum_a x_a\right) \sum_{a_1\neq\ldots\neq a_{n-1}} \prod_{j=1}^{n-1} x_{a_j} \tag{9}$$

If we imagine expanding this as a sum of products of n variables, there will exist two types of terms: those where all x's in the term are unique, and those where x_a from the first sum equals exactly one of the x_{a_j}. Each of the former is overcounted by a factor of n, and each of the latter by a factor of $n-1$:

$$\begin{aligned}(l=1) \qquad \left(\sum_a x_a\right) \sum_{a_1\neq\ldots\neq a_{n-1}} \prod_{j=1}^{n-1} x_{a_j} &= n \sum_{a_1\neq\ldots\neq a_n} \prod_{j=1}^{n} x_{a_j} + (n-1) \sum_{a_1\neq\ldots\neq a_{n-1}} x_{a_1}^2 \prod_{j=2}^{n-1} x_{a_j} \\ &= nC^{(n)} + (n-1) \sum_{a_1\neq\ldots\neq a_{n-1}} x_{a_1}^2 \prod_{j=2}^{n-1} x_{a_j}\end{aligned} \tag{10}$$

The rest of the terms in the l sum of Supplementary Eq. 8 serve solely to cancel out the extraneous terms on the right. We can see that the $l=2$ term splits into two pieces, similar as to how the $l=1$ term did:

$$(l=2) \qquad -\left(\sum_a x_a^2\right) \sum_{a_1\neq\ldots\neq a_{n-2}} \prod_{j=1}^{n-2} x_{a_j} = -(n-1) \sum_{a_1\neq\ldots\neq a_{n-2}} x_{a_1}^2 \prod_{j=2}^{n-2} x_{a_j} - (n-2) \sum_{a_1\neq\ldots\neq a_{n-3}} x_{a_1}^3 \prod_{j=2}^{n-3} x_{a_j} \tag{11}$$

So, adding together the $l=1$ and $l=2$ terms cancels the second sum on the right hand side of Supplementary Eq. 10 which contains terms involving x_a^2, but introduces another extraneous sum of terms involving x_a^3. In general, the lth term expands to

$$(-1)^{l-1}\left((n-l+1) \sum_{a_1\neq\ldots\neq a_{n-l}} x_{a_1}^l \prod_{j=2}^{n-l} x_{a_j} + (n-l) \sum_{a_1\neq\ldots\neq a_{n-l-1}} x_{a_1}^{l+1} \prod_{j=2}^{n-l-1} x_{a_j}\right). \tag{12}$$

From this expansion, we can see that the left piece of the lth summand cancels the right piece of the $l-1^{\text{th}}$ summand. This expansion continues to unzip up until the $l=n$ term, $(-1)^{n-1}\sum_a x_a^n$, in which the right piece is zero. Hence, once the sum is fully unzipped, the only term remaining is the left piece of the $l=1$ summand, which is exactly $C^{(n)}$.

This result still holds for multi-channel images and filters. To restore a channel index, all of the above equations will remain true with the transformations

$$x_a \equiv f_a S_{i+a} \to x_{k,a} \equiv f_{k,a} S_{k,i+a} \qquad \sum_{a_1\neq\cdots\neq a_n} \to \sum_{(a_1,k_1)\neq\cdots\neq(a_n,k_n)} \tag{13}$$

where $k=\{1,2,\ldots K\}$ runs over the number of channels K in the input snapshot.

Using Supplementary Eq. 8, we can compute each of these $C^{(n)}$ in order, efficiently utilizing the results of previous computations to only require $\mathcal{O}(N^2KP)$ operations per site total. The coefficients in parentheses can be seen to be the result of taking the convolution of the lth power of the convolutional filter with the lth power of the occupancy snapshot, taken pixelwise. We can save on an extra bit of computation (though not changing the overall complexity) if the system is fermionic, in which case $S^l = S$ for arbitrary $l \geq 1$.

IV. SUPPLEMENTARY NOTE 4: EXACT MEASUREMENTS

To confirm that our ML models are indeed finding true physical features, we have explicitly calculated correlator estimates for both of the training datasets.

We first measure some examples of "simple" observables at the $\delta = 0.09$ doping level studied in this work. We define $s_{ij} = +1, -1, 0$ if a spin up, spin down, or hole, respectively, lives at site (i,j). We measure the staggered magnetization,

$$m_z = \sum_{i,j} (-1)^{i+j} s_{i,j}, \tag{14}$$

and the sign-corrected nearest neighbor spin-spin correlator,

$$C_s(1) = -\left(\frac{\langle s_{i,j}s_{i+1,j}\rangle + \langle s_{i,j}s_{i,j+1}\rangle - \langle s_{i,j}\rangle\langle s_{i+1,j}\rangle - \langle s_{i,j}\rangle\langle s_{i,j+1}\rangle}{2}\right), \tag{15}$$

with results shown in Supplementary Fig. 8.

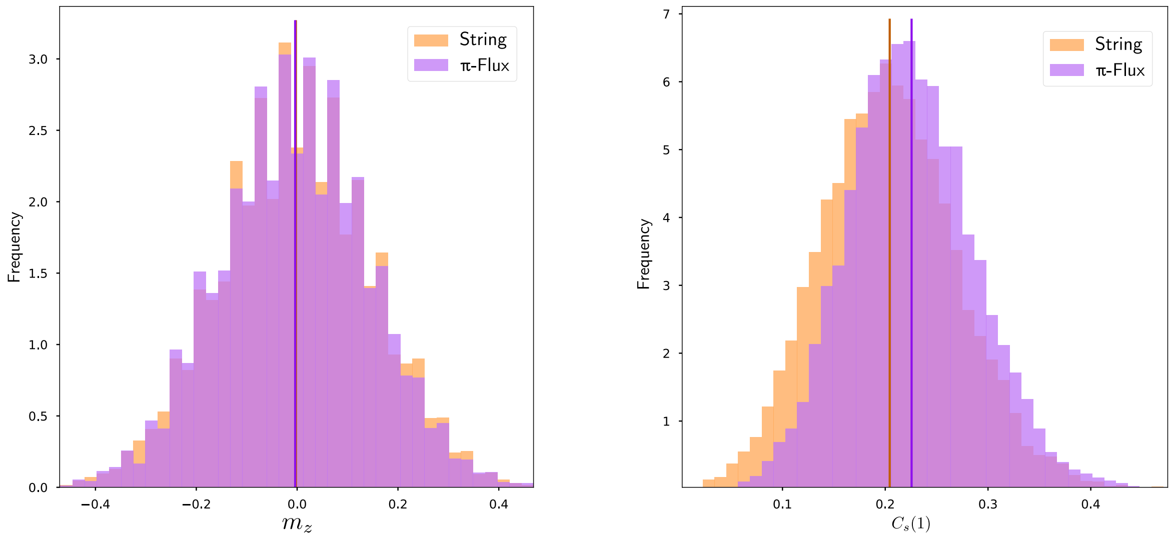

(a) Staggered magnetization.

(b) Nearest-neighbor sign-corrected correlator.

Supplementary Figure 8: **Histograms of "simple" observables measured from single snapshots of each theory**. Each histogram is scaled to integrate to one. Vertical lines denote the mean of each distribution.

We see that the staggered magnetization is nearly indistinguishable, though the nearest-neighbor spin correlator does show some minor deviation between the theories. However, this discrepancy is hardly enough to explain the $> 80\%$ classification accuracy of ours and previous [1] ML models. Indeed, our 2nd-order CCNN can pick up this correlation, and only manages to achieve $\approx 63\%$ classification accuracy.

We now turn to the fourth-order correlations discovered by the CCNN. In Supplementary Fig. 9, we show histograms of correlator estimates obtained from single snapshots contained within the two datasets. Due to the D_4 symmetry of the models, we average over all symmetry-equivalent versions of each correlator for each estimate as the symmetrization of our ML models would. From the figure, we can see that the patterns which are the dominant subpatterns of the learned CCNN filters are indeed biased towards the theory in alignment with what the model predicts, with many distributions being more clearly separated than the two-point NN correlator distributions from Supplementary Fig. 8b. We can also see from the figure that some subpatterns contained in the filters actually show no significant difference between the two theories; our interpretation of this is that these patterns emerge as "connections" when the CCNN attempts to include multiple significant patterns within a single filter. Since these connecting patterns are statistically identical between the two theories, including them is a "free" action to the network which will not hamper performance.

In Supplementary Fig. 10, we plot measured fourth-order correlators obtained from the two datasets as a function of hole doping. While all models in this work are trained on data at 9% doping, this plot shows an interesting trend. We note that at 0% doping, the "parallel spin" correlator (red) is nearly identical between the two theories. It is only once a finite hole doping is introduced that these correlators begin to deviate from each other. This agrees with our explanation of strings leaving a "wake" of parallel spins, increasing this four-site correlator relative to the π-flux theory. While the current network cannot see the connected components of these correlations, we can see in Supplementary Fig. 10(b) that the finite-doping connected component has opposite sign to the zero-doping value, indicating the key role mobile holes play in modifying this purely-spin correlator. All connected correlation functions are computed using the standard formula for the Ursell function of four variables:

$$\begin{aligned}
u_4(A,B,C,D) = \langle ABCD\rangle &- \langle A\rangle\langle BCD\rangle - \langle B\rangle\langle ACD\rangle - \langle C\rangle\langle ABD\rangle - \langle D\rangle\langle ABC\rangle \\
&- \langle AB\rangle\langle CD\rangle - \langle AC\rangle\langle BD\rangle - \langle AD\rangle\langle BC\rangle \\
&+ 2\langle AB\rangle\langle C\rangle\langle D\rangle + 2\langle AC\rangle\langle B\rangle\langle D\rangle + 2\langle AD\rangle\langle B\rangle\langle C\rangle \\
&+ 2\langle BC\rangle\langle A\rangle\langle D\rangle + 2\langle BD\rangle\langle A\rangle\langle C\rangle + 2\langle CD\rangle\langle A\rangle\langle B\rangle \\
&- 6\langle A\rangle\langle B\rangle\langle C\rangle\langle D\rangle, \qquad (16)
\end{aligned}$$

where A, B, C, D are one of $\{\hat{n}_\uparrow, \hat{n}_\downarrow, (1 - \hat{n}_\uparrow - \hat{n}_\downarrow)\}$ operating on different sites of the lattice.

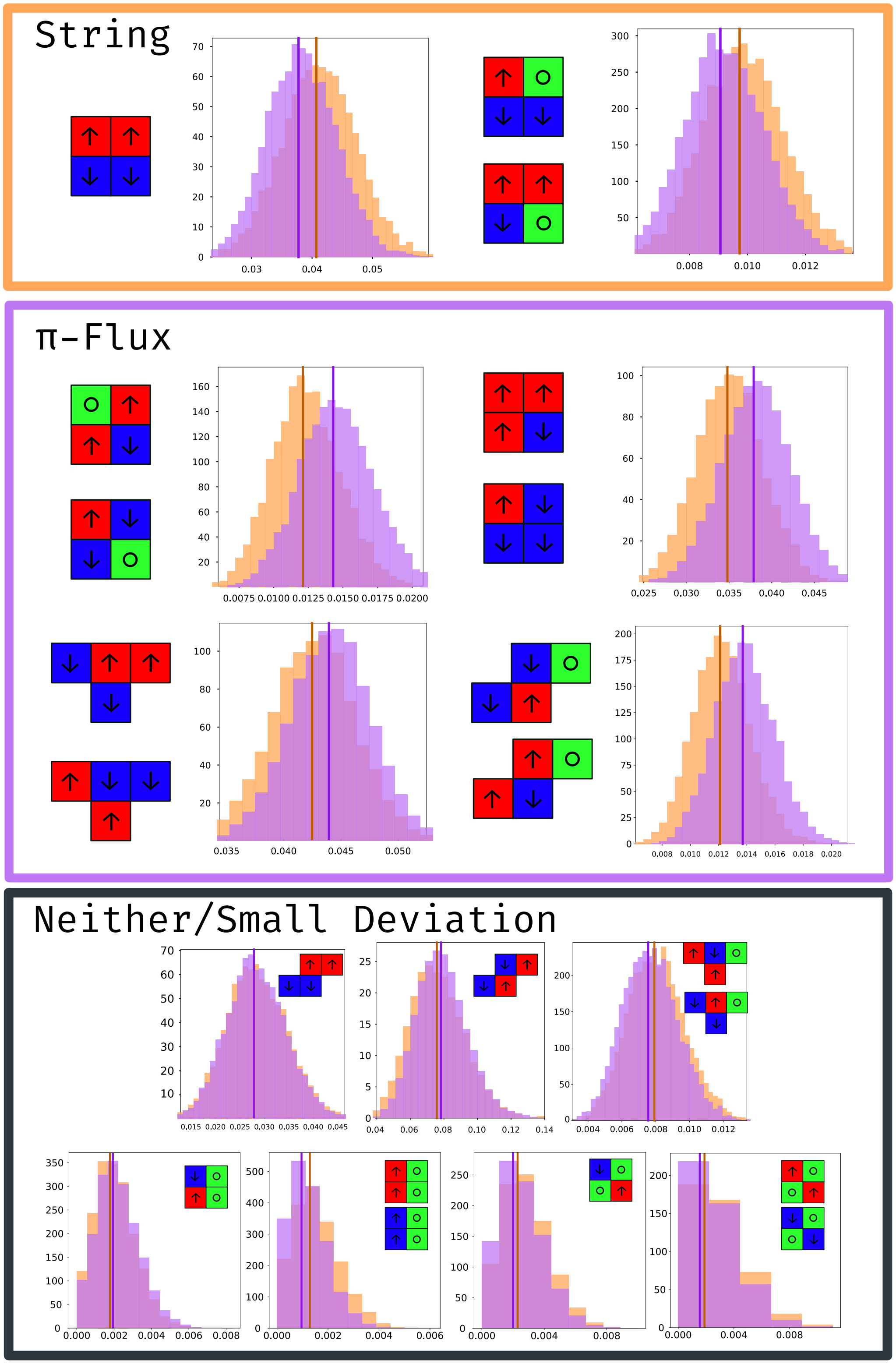

Supplementary Figure 9: **Explicit statistical measurements of various fourth-order bare correlators.** Correlator values are averaged across all patterns symmetry-equivalent under rotations, spatial flips, and spin-flips. Histogrammed are normalized counts of each pattern (and its symmetry equivalents) obtained from single snapshots of each theory, with each histogram scaled to integrate to one. Vertical lines denote the mean of each distribution.

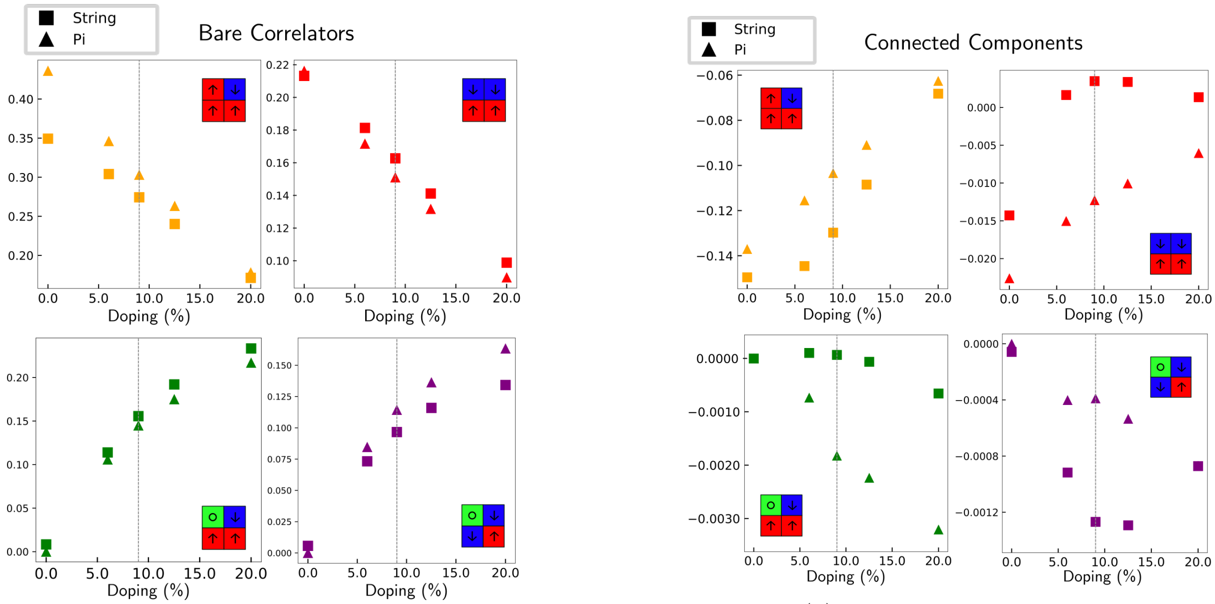

(a) Bare correlations

(b) Connected correlations

Supplementary Figure 10: **Doping dependence of discovered fourth-order correlators.** Correlators are measured from the snapshot datasets of both theories as a function of hole doping summed across all correlators symmetry-equivalent under rotations, spatial flips, and spin-flips. This work trained on data sampled at 9% doping. The CCNN can only see the bare correlations, whose values are shown in (a). The connected components of these correlators, corresponding to the "additional" contributions from true fourth-order effects, are drawn in (b).

V. SUPPLEMENTARY NOTE 5: REGULARIZATION PATHS OF TRADITIONAL CNNS

The regularization path technique demonstrated in the main text can also be applied to shallow traditional CNNs as an alternative to more complex techniques such as Layerwise Relevancy Propagation [15] which are appropriate for deeper models. In these scenarios, regularization paths can still determine which *filters* are contributing most to the classification, however information about which *orders* of correlations are important is inaccessible. We present here an example, and additionally show that fully-connected layers tend to simply "memorize" quantum gas data rather than learn true physics by "reducing" a full CNN architecture to a simpler one with minimal modifications

As a demonstration, consider a CNN with an extremely similar architecture as our CCNN, but using a standard nonlinear step, as seen in Supplementary Fig. 11b. The input S is convolved with a set of learned filters f_α to produce activation maps C_α. Then, a nonlinear function σ is applied pixelwise to the activation map to produce $\tilde{C}_\alpha = \sigma(C_\alpha)$; here we choose σ = relu. These are spatially averaged to produce c_α features, which are used by a final logistic classifier with coefficients β_α. Heuristically, each c_α captures how much the patterns seen in the snapshot S "look like" the filter patterns f_α. Note that a BatchNorm layer is not strictly needed here due to the relatively uniform scale of the features c_α; it may help training progress easier, but is not required for the model to work. Hence, we will omit it for this test. Additionally, we have found that the model becomes extremely difficult to train if the filter parameters f_α are forced positive as we did for CCNNs. As a consequence, we were unable to apply this constraint, resulting in filters which are harder to interpret than for CCNNs.

To perform a "reduction" to this model from the architecture of [1], we first train their architecture exactly as we did for CCNNs: the full model is first trained with an L1 regularization on the filters f_α. After training, the filters are frozen, the fully connected layer is replaced with simple spatial averaging, and the β_α coefficients are regressed at various strengths of regularization applied to them. The result of doing this process can be seen in Supplementary Fig. 11. We observed that the replacement of the fully-connected layer with simple averaging actually improves validation performance as shown in Supplementary Fig. 11, providing empirical evidence that this overparameterized layer is simply "memorizing" the input data rather than learning true physical features. Meanwhile, both the reduced CNN and the CCNN architectures see train and val accuracy track each other well. While it is possible to reduce the train-val gap for the full CNN model using strong regularization techniques, we found it difficult to beat the performance of these small models.

We can attempt to interpret the path in Supplementary Fig. 11(d) by examining the patterns of the filters which

activate first. The first feature to activate, labeled in purple, seems to have something to do with local antiferromagnetic correlations. The second feature, in grey, as well as the blue feature, seem to have something to do with spin-hole correlations. However, it is not as clear how we should understand the red or brown features which activate. Additionally, for each of these patterns we are unable to tell what order feature from the pattern is actually used. The additional complexity of allowing for negative values in filters, along with being unable to disentangle different orders of correlations, make direct interpretation of traditional CNNs generally difficult.

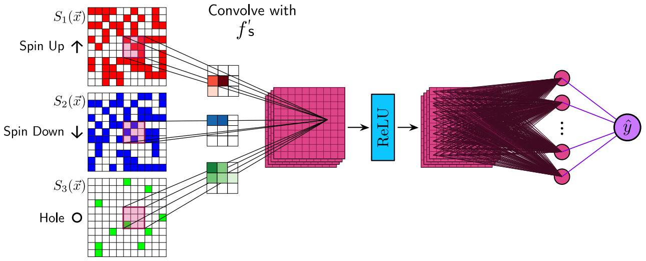

(a) A diagram of the full CNN adapted from [1].

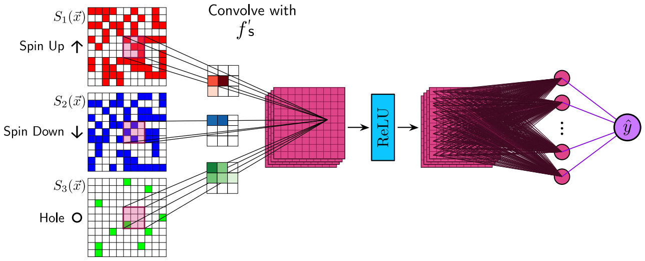

(b) A diagram of the reduced CNN.

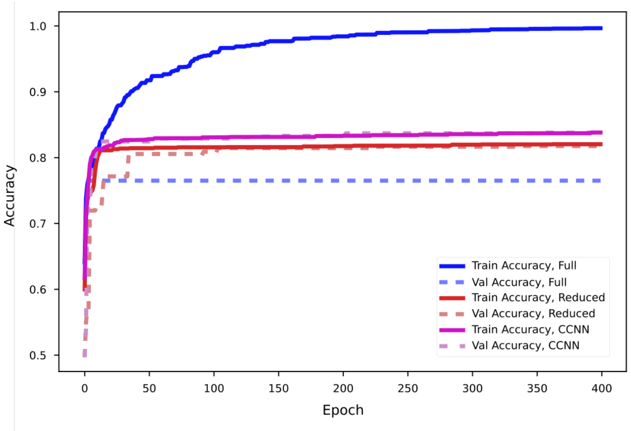

(c) Train and val accuracy comparisons between the full and reduced CNN models, as well as a CCNN model. All accuracies shown are a running max over all previous epochs to smooth out the noisy val performance. All models have two filters, and have an L1 penalty of $\gamma = 0.005$.

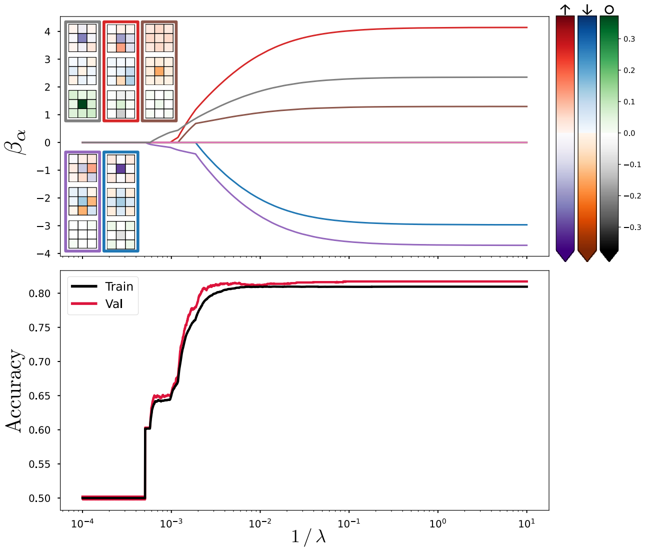

(d) Regularization path obtained from a trained reduced CNN.

Supplementary Figure 11: **Attempting a regularization path procedure on a standard CNN architecture.**

VI. SUPPLEMENTARY NOTE 6: "DEBUGGING" TRIVIAL BEHAVIORS

As a demonstration of a case where CCNNs can be helpful in detecting "trivial" features of the data, we will train a CCNN to distinguish between real experimental data (from [2]) and simulated π-flux snapshots, both at 9% doping. We tune the temperature of the π-flux simulation as to best match the nearest-neighbor spin correlator with experimental data, as done in [1]. The snapshots are zero-masked to the same geometry of the experiment. Because our QGM experiments currently cannot resolve both spin species simultaneously, we convert all spin-down sites in the π-flux snapshots to appear as empty sites. Additionally, double-hole pairs also appear as empty sites in the experiment due to a parity projection; to accomodate this we insert doublon-hole pairs randomly on neighboring sites in the lattice with a probability matching the theoretical predictions, also appearing as empty sites. As we have two species of site (spin up and "empty"), this results in a set of two-channel snapshots input to the network.

We only have access to a small amount of experimental data, meanwhile π-flux data is essentially limitless. However, if we naively train a ML model with a dataset that contains significantly more data of one class than the other, a significant local minima the network can be trapped in is to simply predict the more populous class all the time. For example, in our dataset we have 2476 experimental snapshots and 19500 π-flux snapshots, so a model which predicts π-flux all the time would achieve an 89% accuracy. A simple solution to this is to *oversample* the experimental snapshots. On each epoch, we randomly duplicate snapshots from the experimental dataset until there are 19500 in the dataset.

As in the main text, we train a CCNN with two filters, and with an L1 loss applied to the filter weights. We use a slightly stronger value of $\lambda = 0.01$, and this was found to not significantly reduce final accuracy compared to the no-regularization case. (Note that the accuracy is much lower here mainly due to the smaller spatial extent of the snapshot: the circular region accessed by experiment contains only 90 sites while the snapshots in the main text contain $15 \times 15 = 225$). The final filters learned are shown in Supplementary Fig. 12a. We can see that the L1 loss has completely turned off one of the filters, while the other still has a collection of pixels left on. Further increasing the L1 loss will turn off more pixels while not affecting the performance, but we stay at this value to demonstrate how the separation of orders in the model allows for identification of trivial behavior.

We might originally guess that this is looking for specific patterns in the snapshots that somewhat resemble the pattern of the filter, i.e. correlators which are subpatterns. However, once constructing the regularization path from the model, shown in Supplementary Fig. 12b, we can see this is not the case! The 1st order correlator explains essentially all of the network's performance, meaning the network is really just measuring the "filled/empty site" occupancy. (In fact, we can see in this instance the higher-order correlations contribute to overfitting, as the validation precision drops when they activate). A simple explanation of this is that the doublon-hole density in the experiment must be higher than expected for the given temperature, and the CCNN can solely use this to tell the snapshots apart.

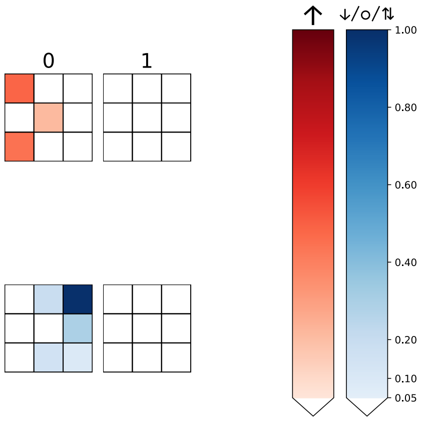

(a) Filters learned from a model trained to classify experimental v. π-flux data

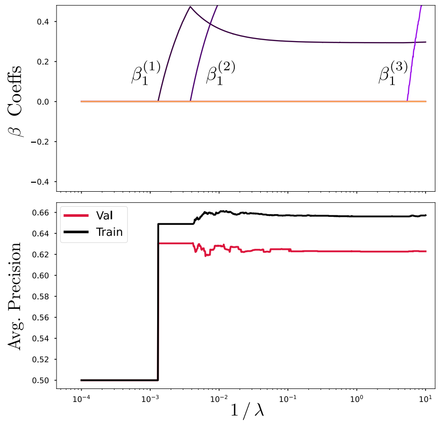

(b) Regularization path related to filters shown in (a). Positive coefficients correspond to π-flux snapshots, negative correspond to experiment.

Supplementary Figure 12: **Model behavior when classifying experimental v. π-flux data.** Here, due to the class imbalance, we plot average precision rather than accuracy in the regularization path, which can be thought of as the average of the two accuracies on the classes individually.

[1] Bohrdt, A. *et al.* Classifying snapshots of the doped Hubbard model with machine learning. *Nature Physics* **15**, 921–924 (2019).
[2] Chiu, C. S. *et al.* String patterns in the doped Hubbard model. *Science* **365**, 251–256 (2019).
[3] Grusdt, F., Bohrdt, A. & Demler, E. Microscopic spinon-chargon theory of magnetic polarons in the tj model. *Physical Review B* **99**, 224422 (2019).
[4] Baskaran, G., Zou, Z. & Anderson, P. The resonating valence bond state and high-Tc superconductivity — A mean field theory. *Solid State Communications* **63**, 973–976 (1987).
[5] Gros, C. Physics of projected wavefunctions. *Annals of Physics* **189**, 53–88 (1989).
[6] Paszke, A. *et al.* PyTorch: An Imperative Style, High-Performance Deep Learning Library. In Wallach, H. *et al.* (eds.) *Advances in Neural Information Processing Systems 32*, 8024–8035 (Curran Associates, Inc., 2019).
[7] Pedregosa, F. *et al.* Scikit-learn: Machine Learning in Python. *Journal of Machine Learning Research* **12**, 2825–2830 (2011).
[8] Tibshirani, R. Regression shrinkage and selection via the lasso: A retrospective. *Journal of the Royal Statistical Society* **73**, 273–282 (2011).
[9] Tibshirani, R., Johnstone, I., Hastie, T. & Efron, B. Least angle regression. *The Annals of Statistics* **32**, 407–499 (2004).
[10] Zou, H. & Hastie, T. Regularization and variable selection via the elastic net. *Journal of the Royal Statistical Society: Series B (Statistical Methodology)* **67**, 301–320 (2005).
[11] Ioffe, S. & Szegedy, C. Batch Normalization: Accelerating Deep Network Training by Reducing Internal Covariate Shift. *arXiv:1502.03167* (2015).
[12] van Laarhoven, T. L2 Regularization versus Batch and Weight Normalization. *arXiv:1706.05350 [cs, stat]* (2017).
[13] Dieleman, S., De Fauw, J. & Kavukcuoglu, K. Exploiting Cyclic Symmetry in Convolutional Neural Networks. *arXiv:1602.02660 [cs]* (2016).
[14] Shalev-Shwartz, S. & Ben-David, S. *Understanding machine learning: from theory to algorithms* (2014).
[15] Bach, S. *et al.* On Pixel-Wise Explanations for Non-Linear Classifier Decisions by Layer-Wise Relevance Propagation. *PLOS ONE* **10**, e0130140 (2015).
